# Lung Function and Gene Expression of Pathogen Recognition Pathway Receptors: the Cardia Lung Study

**DOI:** 10.1038/s41598-020-65923-z

**Published:** 2020-06-09

**Authors:** Ramya Ramasubramanian, Ravi Kalhan, David R. Jacobs, George R. Washko, Lifang Hou, Myron D. Gross, Weihua Guan, Bharat Thyagarajan

**Affiliations:** 1 Division of Epidemiology and Community Health, University of Minnesota School of Public Health, Minneapolis, MN USA; 2 Department of Preventive Medicine, Northwestern University Feinberg School of Medicine, Chicago, IL USA; 3 Division of Pulmonary and Critical Care Medicine, Northwestern University Feinberg School of Medicine, Chicago, IL USA; 4 Division of Pulmonary and Critical Care Medicine, Brigham and Women’s Hospital, Boston, MA USA; 5 Applied Chest Imaging Laboratory, Brigham and Women’s Hospital, Boston, MA USA; 6 Department of Pathology and Laboratory Medicine, University of Minnesota School of Medicine, Minneapolis, MN USA; 7 Department of Biostatistics, University of Minnesota School of Public Health, Minneapolis, MN USA

**Keywords:** Toll-like receptors, Risk factors

## Abstract

Activation of toll-like receptors (*TLR1, TLR5, TLR6*) and downstream markers (*CCR1, MAPK14, ICAM1*) leads to increased systemic inflammation. Our objective was to study the association between the gene expression levels of these six genes and lung function (Forced Expiratory Volume in one second (FEV_1_), Forced Vital Capacity (FVC) and FEV_1_/FVC). We studied gene expression levels and lung function in the Coronary Artery Risk Development in Young Adults study. Spirometry testing was used to measure lung function and gene expression levels were measured using the Nanostring platform. Multivariate linear regression models were used to study the association between lung function measured at year 30, 10-year decline from year 20 to year 30, and gene expression levels (highest quartile divided into two levels – 75th to 95th and>95th to 100th percentile) adjusting for center, smoking and BMI, measured at year 25. Year 30 FEV_1_ and FVC were lower in the highest level of *TLR5* compared to the lowest quartile with difference of 4.00% (p for trend: 0.04) and 3.90% (p for trend: 0.05), respectively. The 10-year decline of FEV_1_ was faster in the highest level of *CCR1* as compared to the lowest quartile with a difference of 1.69% (p for trend: 0.01). There was no association between gene expression and FEV_1_/FVC. Higher gene expression levels in *TLR5* and *CCR1* are associated with lower lung function and faster decline in FEV_1_ over 10 years, in a threshold manner, providing new insights into the role of inflammation in lung function.

## Introduction

Biomarkers of systemic inflammation such as C-reactive protein (CRP) and fibrinogen have been associated with lower FEV_1_ and FVC, chronic obstructive pulmonary disease (COPD) and asthma^1–6^. However, these are non-specific inflammatory biomarkers that do not help elucidate the underlying biological pathways that increase systemic inflammation. For example, CRP can be modulated by numerous biological pathways such as NF-κB or the JAK/STAT pathway^7,8^ and activation of any one of these pathways would increase CRP levels. Thus, measuring CRP alone does not identify specific causes of inflammation. A better understanding of specific biological pathways that modulate down-stream inflammatory markers such as CRP may be useful in understanding the pathogenesis of pulmonary diseases such as COPD and asthma. Technological advances have enabled measurement of gene expression levels for thousands of targets simultaneously in epidemiological studies^9,10^ and can provide novel insights into biological pathways that influence the pathogenesis of chronic diseases.

In this study, we evaluated the pathogen recognition pathway, which is a specific inflammatory pathway, mediated by Toll-Like Receptors (TLRs), which leads to the activation and nuclear translocation of NF-κB that promotes inflammation. TLRs are activated after they specifically recognize Pathogen Associated Molecular Patterns (PAMPs) from a variety of pathogens^11,12^ and are involved in the pathogenesis of inflammatory diseases such as asthma and COPD^13,14^. *TLR1* and *TLR6* are cell surface receptors found on the surface of innate immune cells such as monocytes, myeloid dendritic cells and plasmacytoid dendritic cells, which form heterodimers with *TLR2* after activation by bacterial lipopeptides from gram positive bacteria^15,16^. *TLR1/TLR2* heterodimer regulates the expression of *CCR1*, a chemokine receptor, which upon activation by its ligands such as *CCL3*, *CCL5*, *CCL7* and *CCL23* helps in the recruitment of immune cells to the site of inflammation^17,18^. *TLR1/TLR2* heterodimer also activates *ICAM1*, which was previously shown to be associated with lung function^19,20^*. TLR5* is another cell surface toll-like receptor which is activated by flagellin, a principal component of bacterial flagellum. *TLR5* is present on the surface of monocytes and myeloid dendritic cells^21,22^ and is involved in the priming of allergic responses to dust allergens promoting asthma^23^. *TLR5*, *TLR6* and *TLR2* activation causes downstream activation of *MAPK14* and the NF-κB pathway^24,25^ that ultimately results in the release of proinflammatory cytokines such as IL-1β, TNF-α and IL-6 and, proliferation of immune cells^26^.

The primary objective of this study was to evaluate associations between gene expression levels, in mixed white cells in a community-based sample, of six inflammatory markers and lung function defined by FEV_1_, FVC and FEV_1_/FVC ratio in the Coronary Artery Risk Development in Young Adults (CARDIA) study. We hypothesized that higher gene expression level of biomarkers associated with increased inflammation would be associated with a lower lung function measurement, and with a faster decline in lung function.

## Methods

### Study population

CARDIA is a cohort study with 5115 participants who were recruited at baseline examination during the year 1985-1986 at 4 field centers (Birmingham,AL: Chicago, IL; Minneapolis, MN; and Oakland, CA). The study included approximately equal number of blacks and white; men and women. The follow-up in 2005–2006 (year 20 exam) included 3547 participants (72% of survivors); in 2010–2011 (year 25 exam) was 3499 (72% of survivors) and in 2015–2016 (year 30 exam) was 3358 (71% of survivors). The detailed methods, instruments and quality control procedures for the CARDIA study have been previously described^27,28^. Demographics, lifestyle habits, physical activity were self-reported using questionnaires. All study methods were carried out in accordance with relevant guidelines and regulations. The CARDIA study is reviewed annually and approved by the internal review boards at Kaiser Permanente Division of Research, Northwestern University, University of Minnesota and University of Alabama at Birmingham. All CARDIA participants provided a signed informed consent before study participation and sign a new informed consent form at every examination.

Three thousand five hundred and forty participants attended the year 20 examination, 3499 participants attended the year 25 examination and 3358 participants attended the year 30 examination. For the cross-sectional analyses using year 25 gene expression levels and year 30 lung function measurements, 2527 participants were included in the analyses after excluding pregnant women (n = 10), participants with missing year 30 lung function data (n = 185), year 25 gene expression measurements (n = 425) and other covariates at year 25 (n = 9 for BMI and n = 54 for smoking). To evaluate association of 10-year decline of lung function from year 20 to year 30 with year 25 gene expression measurements, 2271 participating were included in the analyses after excluding an additional 117 participants with missing lung function data at year 20 in addition to the participants excluded in the cross-sectional analyses. The participants with lung function data were more likely to have lower BMI (29.92 vs. 30.64; p-value = 0.005), lower alcohol consumption (10.91 mL per day vs. 13.22 mL per day; p-value = 0.01), lower C-reactive protein (2.96 vs. 3.84; p-value = 0.0007), had lower percentage of blacks (42.89% vs. 58.49%; p-value <0.0001), had higher percentage of women (58.43% vs. 51.35%; p-value <0.0001) and lower current smokers (12.86% vs. 25.30%; p-value <0.0001) as compared to CARDIA participants who were not included in the analyses (n = 2844). For the sensitivity analysis, we excluded 55 participants with COPD and 476 participants with asthma in years 25 and 30 when evaluating the cross-sectional association between year 30 lung function and year 25 gene expression. We also excluded 47 participants with COPD and 442 participants with asthma at years 20, 25 and 30 to evaluate the longitudinal association between 10-year decline of lung function and year 25 gene expression.

### Spirometry

Spirometry was performed using a dry rolling-seal OMI spirometer (Viasys Corp, Loma Linda, CA) at year 20 examination and a portable spirometer EasyOne Diagnostic, NDD Medical Technologies, Andover,MA) at year 30 following the American Thoracic Society Guidelines^29^. Daily checks for leaks, volume calibration with a 3-liter syringe and weekly calibration in the 4–7 litre range were undertaken to minimize methodological artifacts between exams. We analyzed FVC and FEV_1_ as the maximum of five satisfactory maneuvers and represented as percent of predicted^30^. In almost all cases, the maximum and second highest maneuvers agreed to within 150 ml.

### Gene expression analysis

Whole blood was collected in the PAXgene Blood RNA tubes (Qiagen Inc., Germantown, MD) at the year 25 examination. mRNA was isolated using the PAXgene Blood RNA kit (Qiagen Inc., Germantown, MD) at the Molecular Epidemiology and Biomarker Research Laboratory (MEBRL) according to the manufacturer’s instructions.

The nCounter analysis system (Nanostring Inc., Seattle, WA) was used to measure gene expression levels using mRNA obtained from whole blood collected in a PAXgene tube. The nCounter system utilized two unique~50 base probes, or “code set” per mRNA, a Capture probe that immobilized the probe/target complex to the nCounter cartridge and a Reporter probe for signal detection. The digitally captured color codes were counted and tabulated for each target molecule. As a quality control measure internal control (positive and negative) code sets were used. The positive control probes are mixed in a varying concentration corresponding to expression levels of most mRNAs of interest, and the negative control probes are used to estimate the non-specific background in the experiment.

Normalization of the gene expression was done with a combination of positive control normalization and CodeSet Content Normalization using housekeeping genes to correct major sources of error including pipetting errors, instrument scan resolution, batch variations and sample input variability. The positive control normalization factor was the ratio of the average ERCC positive control intensity seen across all samples divided by the positive control within an individual sample. The geometric mean of the 5 housekeeping genes (*B2M*, *GAPDH*, *GNB1*, *HPRT1*, *PGK1*) was calculated for each sample and the average geometric mean across all samples divided by the geometric mean of the housekeeping genes for each sample was used to normalize gene expression levels. In addition, the average count for each target is calculated using all the samples probed within a particular CodeSet and this average is used to calculate a CodeSet normalization factor to account for the variation in the efficiency at capturing and scanning each unique target type. The raw counts of the gene expression of sample were first multiplied by the sample specific positive control normalization factor, then by the housekeeping gene normalization factor and the CodeSet normalization factor to obtain the final gene expression counts.

### Measurement of covariates and determination of asthma/COPD status

Smoking status was measured using questionnaires. Never smokers were defined as participants who reported never having used any tobacco products such as cigarettes, cigars or not having smoked regularly for at least three months. Former smokers were defined as having smoked regularly for at least three months but are not currently smoking regularly. Current smokers were defined as having smoked regularly for at least three months and are smoking regularly currently. BMI was calculated using the height and weight variables as weight (in kg) divided by height (in meters) squared. BMI and smoking status measured at year 25 were used in the analysis. Asthma was defined by self-report of whether the participants were ever diagnosed as having asthma or if they were currently taking any asthma medications. COPD was also self-reported by the participants.

### Statistical methods

Selected year 25 characteristics among 5 levels of *TLR5* gene expression were compared using chi-square tests for categorical variables and one-way ANOVA for continuous variables. Normalized gene expression counts of 16 was set as the lower limit of detection and signal lower than the LLD was set at 16 prior to data analysis. Distribution of the gene expression of the genes was studied with scatterplots. The gene expression of *TLR1, TLR6, TLR5*, *MAPK14, CCR1* and *ICAM1* were divided into quartiles and the highest quartile was divided into two levels: 75th to 95th percentile and>95th to 100th percentile (the top twentieth). We evaluated the association between lung function at CARDIA exam years 30, 10-year decline in lung function (year 20-year 30) and year 25 gene expression levels of *TLR5, MAPK14* and *CCR1* using linear regression models after adjustment for center, smoking status (never, former, current smokers at year 25) and body mass index (BMI) (year 25). We defined percent predicted lung function as the ratio of observed lung function over predicted lung function, where predicted lung function was calculated using the Hankinson equation^30^. We created an inflammation score by adding the quartile levels of *TLR5*, *MAPK14* and *CCR1* for each participant so that the maximum inflammation score would be 15 when all markers were in the highest level (top twentieth) while the minimum inflammation would be 3 when all markers were in the first quartile. We also created an inflammation score using quartile levels of the six genes such that the maximum inflammation score is 30 when the six genes are in the highest level and the minimum inflammation score is 6 when the six genes are in the first quartile. Multivariable linear regression models were used for evaluating the association between lung function at CARDIA exam year 30, 10-year decline in lung function and inflammation score as a continuous predictor variable. Sensitivity analysis was performed by excluding asthma and COPD patients at CARDIA exam years 20 and 30, and evaluation of the association between lung function at CARDIA exam year 30, 10- year decline in lung function and year 25 gene expression levels of *TLR1, TLR5, TLR6, MAPK14*,*CCR1, ICAM1* in the subgroup of participants without COPD/asthma. All the p-values ≤ 0.05 were considered statistically significant. Statistical analyses were carried out using SAS software version 9.4 (SAS Institute, Cary, NC).

## Results

### Characteristics at year 25 examination

The participants in highest level (top twentieth) of *TLR5* at year 25 were more likely to be black (57.52% vs 44.64%; p-value = 0.0006), current smokers (23.01% vs. 8.80%; p < 0.0001), have higher BMI (34.34 kg/m^2^ vs. 28.66 kg/m^2^; p < 0.0001) and have higher C-reactive protein (CRP) (7.79 µG/mL vs. 1.69 µG/mL; p < 0.0001) as compared to those in the first quartile of *TLR5* (Table 1).There were no consistent differences in age and alcohol consumption among the five levels of *TLR5*. In general, participants with the highest levels of other genes such as *CCR1*, *TLR1*, *TLR6*, and *ICAM1* showed similar distribution as *TLR5* with participants being more likely to be black, women, have higher BMI and higher CRP as compared to the respective genes’ first quartiles, except for *MAPK14*, where the participants in the highest level of *MAPK14* were more likely to be white (63.72% vs. 39.08%; p-value <0.001). (Supplementary Tables 1a-1e). The scatterplots of the gene expressions showed a wide range of the distribution of the gene expression levels for all six genes, with and highest correlation seen between *TLR1* and *TLR6* (r = 0.73; p- <0.01). In general, there was a higher correlation between the TLR genes (*TLR1*, *TLR5* and *TLR6*) (r = 0.49–0.73; p < 0.01) while there were only modest correlations seen between TLR genes and genes in downstream pathways such as *CCR1* and *ICAM1* (r = 0.23–0.33; p < 0.01). However, the associations between *TLR* genes and *MAPK14* were stronger (r = 0.54–0.64; p < 0.01) (Supplementary Fig. 1).

**Table 1:** Participant characteristics at year 25 with respect to *TLR5* gene expression levels.

Characteristics	*TLR5* gene expression levels					p-value
	0–25 percentile (n = 569)	>25–50 percentile (n = 567)	>50–75 percentile (n = 568)	>75–95 percentile (n = 454)	>95–100 percentile (n = 113)	
Age (years)	50.25 (3.57)	50.30 (3.52)	50.27 (3.55)	49.74 (3.64)	49.79 (3.64)	0.06
Race						
%Blacks	44.64	38.45	39.79	46.48	57.52	0.0006
Sex						
% Female	59.93	54.14	56.16	64.32	60.18	0.01
Smoking						
Never	67.49	68.96	65.32	58.59	53.10	<0.0001
Former	23.55	22.75	19.37	23.35	24.78	
Current	8.96	8.29	15.32	18.06	22.12	
BMI	28.66 (6.18)	28.94 (6.38)	29.86 (6.62)	31.67 (7.67)	34.27 (10.09)	<0.0001
Alcohol consumption (mL/day)	9.24 (15.88)	11.59 (23.31)	11.92 (19.41)	10.79 (19.98)	11.26 (23.76)	0.19
C-reactive protein (uG/ML)	1.69 (2.44)	2.14 (3.38)	2.81 (3.80)	4.58 (6.23)	7.79 (9.62)	<0.0001

### Association between year 30 lung function and year 25 gene expression profiles

Year 30 predicted FEV_1_ and FVC was lower in the highest level (top twentieth) of *TLR5* as compared to the lowest quartile of *TLR5*, with a difference of 4.00% (95% CI: 0.95–7.05; p for trend: 0.04) and 3.90% (95% CI: 1.14–6.65; p for trend: 0.05), respectively. (Table 2). None of the other genes were associated with year 30 FEV_1_ or FVC (Table 2, Supplementary Table 2). Inflammation score was associated with year 30 predicted FEV_1_ and FVC. The difference in FEV_1_ between the highest inflammation score (for *TLR5, MAPK14* and *CCR1*) and lowest inflammation score was 6.21 (95% CI: −2.25, 14.68; p = 0.02) while difference in FVC was 5.34 (95% CI: −2.34, 13.02; p = 0.03). Year 30 predicted FEV_1_/FVC and the inflammation score were not associated with FEV_1_/FVC (Table 2, Supplementary Table 2). The inflammation score using six genes was not associated with year 30 FEV_1_, FVC and FEV_1_/FVC ratio. The difference between year 30 predicted FEV_1_ and FVC at maximum inflammation score compared to minimum inflammation score was 8.68% (95% CI: −13.81, 31.18) and 9.87 (95% CI: −10.46, 30.19), respectively.

**Table 2:** Association between year 30 lung function and year 25 gene expression levels.

Markers	0 to 25 percentile	25 to 50 percentile	50 to 75 percentile	75 to 95 percentile	95 to 100 percentile	Difference between lowest quartile and highest levels	p-value for trend
**Year 30% predicted FEV**_**1**_							
*TLR5*	92.54 ± 0.63	93.77 ± 0.63	92.10 ± 0.62	92.03 ± 0.70	88.54 ± 1.41	4.00 (0.95, 7.05)	0.04
*MAPK14*	92.54 ± 0.62	92.81 ± 0.62	92.60 ± 0.62	92.16 ± 0.70	90.32 ± 1.41	2.22 (−0.80, 5.24)	0.29
*CCR1*	93.43 ± 0.63	91.57 ± 0.62	92.13 ± 0.62	92.23 ± 0.70	94.11 ± 1.40	−0.68 (−3.70, 2.34)	0.45
**Year 30% predicted FVC**							
*TLR5*	94.35 ± 0.57	95.27 ± 0.57	93.72 ± 0.56	94.15 ± 0.64	90.45 ± 1.28	3.90 (1.14, 6.65)	0.05
*MAPK14*	94.39 ± 0.56	94.46 ± 0.56	94.06 ± 0.56	93.98 ± 0.63	93.26 ± 1.27	1.13 (−1.60, 3.86)	0.27
*CCR1*	94.77 ± 0.57	93.97 ± 0.56	93.82 ± 0.57	94.02 ± 0.63	94.84 ± 1.27	−0.07 (−2.81, 2.66)	0.30
**Year 30% predicted FEV**_**1**_**/ FVC**							
*TLR5*	98.18 ± 0.34	98.38 ± 0.34	98.35 ± 0.34	97.80 ± 0.38	98.88 ± 0.76	0.30 (−1.34, 1.94)	0.50
*MAPK14*	98.08 ± 0.34	98.27 ± 0.34	98.46 ± 0.34	98.19 ± 0.38	96.82 ± 0.75	1.27 (−0.36, 2.89)	0.97
*CCR1*	98.69 ± 0.34	97.52 ± 0.34	98.15 ± 0.34	98.07 ± 0.38	99.55 ± 0.75	−0.86 (−2.48, 0.76)	0.79

Note: All percent predicted estimates are represented as percentage ± SE. The differences are represented with the 95% CI. Linear regressions with adjustment for center and year 25 smoking (never, former, current). 2527 participants included in the analysis.

### Association between 10-year decline in lung function and year 25 gene expression profiles

Decline in FEV_1_, FVC and FEV_1_/FVC from year 20 to year 30 was not significantly associated with year 25 *TLR5* expression levels (Table 3). Contrary to our hypothesis, decline in FEV_1_ and FVC was lower in the highest level (top twentieth) of *MAPK14* as compared to the lowest quartile (1.60% vs 3.19%; p for trend =0.03 and 1.86% vs 3.73%; p for trend=0.008). Decline in FEV_1_/FVC was not significantly associated with *MAPK14* expression levels. Consistent with our hypothesis, decline in FEV_1_ was significantly higher in the highest level of *CCR1* as compared to the lowest quartile (3.43% vs. 1.73%; p for trend=0.01) (Table 3). Decline in FVC and FEV_1_/FVC was not significantly associated with *CCR1*. Expression levels of other genes were not associated with 10-year decline in FVC, FEV_1_ and FEV_1_/FVC (Supplementary Table 3). The inflammation score of *TLR5*, *CCR1* and *MAPK14* was not associated with the 10-year decline in FEV_1_, FVC and the FEV_1_/FVC ratio (difference in 10 year decline in FEV_1_ between highest and lowest inflammation scores = 1.11% (95% CI: −3.95,6.16; p = 0.83), difference in 10 year decline in FVC between highest and lowest inflammation scores = −0.85 (95% CI: −5.86, 4.16; p = 0.52), difference in 10 year decline in FEV_1_/ FVC between highest and lowest inflammation scores = 2.03 (95% CI: −0.88, 4.94; p = 0.39). The inflammation score created using six genes was not associated with 10-year decline of FEV_1,_ FVC and FEV_1_/FVC ratio. The difference between 10-year decline of predicted FEV_1_ and FVC at maximum inflammation score compared to minimum inflammation score was −5.53 (95% CI: −24.11, 13.05) and −9.33 (95% CI: −27.74, 9.07), respectively.

**Table 3:** Association between 10-year change in lung function from year 20 to year 30 and year 25 gene expression profiles.

Markers	0 to 25 percentile		25 to 50 percentile		50 to 75 percentile		75 to 95 percentile		95 to 100 percentile	Difference between lowest quartile and highest levels	p-value for trend
**% predicted FEV**_**1**_**–10-year decline**											
*TLR5*		2.74±0.39		2.32±0.39		2.18±0.39		2.79±0.44	3.52±0.89	−0.69 (−2.59, 1.22)	0.94
*MAPK14*		3.19±0.39		2.55±0.39		2.37±0.39		2.19±0.44	1.60±0.88	1.58 (−0.29, 3.47)	0.03
*CCR1*		1.73±0.39		2.66±0.39		2.83±0.39		2.83±0.44	3.43±0.88	−1.69 (−3.59, 0.19)	0.01
**% predicted FVC – 10-year decline**											
*TLR5*		3.19±0.39		2.78±0.39		2.59±0.39		2.98±0.44	3.45±0.88	−0.25 (−2.15, 1.65)	0.78
*MAPK14*		3.73±0.39		2.93±0.39		2.72±0.39		2.36±0.43	1.86±0.87	1.86 (−0.01, 3.72)	0.008
*CCR1*		2.61±0.39		2.72±0.39		3.06±0.39		3.00±0.43	4.25±0.87	−1.64 (−3.51, 0.23)	0.06
**% predicted FEV**_**1**_**/ FVC – 10-year decline**											
*TLR5*		−1.16±0.22		−0.90±0.23		−0.99±0.22		−0.83±0.25	−0.35±0.51	−0.81 (−1.91, 0.29)	0.38
*MAPK14*		−1.04±0.22		−0.98±0.22		−0.96±0.22		−0.79±0.25	−0.86±0.50	−0.18 (−1.27, 0.90)	0.66
*CCR1*		−1.31±0.23		−0.53±0.22		−0.87±0.22		−0.86±0.25	−1.97±0.50	0.66 (−0.42, 1.74)	0.87

Note: All percent predicted estimates are represented as percentage ± SD. The differences are represented with the 95% CI. Linear regressions with adjustment for center and year 25 smoking (never, former, current). 2271 participants included in the analysis.

### Sensitivity analysis after exclusion of asthma and COPD patients

The distribution of asthma and COPD participants was similar across all quartiles of *MAP14* (24.29% vs. 20.37%; p = 0.32 and 3.06% vs. 2.43%; p = 0.82), *CCR1* (24.66% vs. 19.10%; p = 0.07 for asthma and 3.29% vs 2.71%; p = 0.49 for COPD). The distribution across *TLR5* quartiles was different (26.30% vs. 20.03%; p = 0.02 for asthma and 4.33% vs. 1.52% for COPD; p = 0.01). After removal of the asthma and COPD patients, we found results similar to those seen in the entire dataset. Briefly, year 30 FEV_1_ and FVC was lower in the highest level (top twentieth) of *TLR5* compared to the lowest level, with a difference of 4.84 (95% CI: 1.68–8.01; p for trend: 0.05) and 4.83 (95%CI: 1.87–7.78; p for trend: 0.03), respectively. The 10-year decline of FEV_1_ was higher in the highest level of *CCR1* as compared to the lowest level with as difference of 2.76 (95% CI: 0.68–4.83; p for trend: 0.02). The 10-year decline of FEV_1_ and FVC was lower in the highest level of *MAPK14* as compared to lowest level with a difference of 1.63% (95% CI: −0.41–3.69; p for trend: 0.01) and 2.01% (95% CI: −0.06–4.08; p for trend: 0.006).

## Discussion

This study showed that higher expression of *TLR5* was associated with lower year 30 predicted FEV_1_ and FVC. In addition, faster 10-year decline of FEV_1_ from year 20 to year 30 was associated with higher gene expression levels of *CCR1*. However, the faster 10-year decline in predicted FEV_1_ and FVC was associated with lower year 25 gene expression levels of *MAPK14*. These results are consistent with the hypothesis that higher gene expression levels of genes in the pathogen recognition pathways were associated with lower lung function for the most part.

Previous studies on inflammation and lung function have utilized non-specific markers of inflammation^2,4,31,32^ and, to our knowledge, this is the first study to evaluate and demonstrate an inverse association between gene expression levels in the pathogen recognition pathway and lung function. A study done with 531 participants from the European Community Respiratory Health Survey found a cross sectional negative relationship between percent predicted FEV_1_ and serum CRP concentration after dividing them into tertiles (p-value = 0.002). Other studies which used markers of systemic inflammation such as fibrinogen also reported a faster lung function decline was associated with higher fibrinogen levels^31,33^. With respect to longitudinal relationship, there have been mixed findings with CRP. A previous study in CARDIA reported a faster decline in FEV_1_ and FVC from year 5 to year 20 among participants in the highest quartile of year 7 CRP compared to the lowest quartile^4^. The study conducted with participants from the European Community Respiratory Health Survey also found that increase in CRP levels was associated with greater mean annual FEV_1_ decline after adjustment for potential confounders (p-value = 0.002)^34^. However, another community based cohort with 2442 individuals found that the baseline CRP value was not associated with FEV_1_ and FVC decline over 9 years^32^. These studies suggest that an overall increase in inflammation is associated with reduced lung function but do not indicate a specific pathway leading to this increase.

Results observed in our study suggest that higher levels of gene expression of a specific inflammatory pathway, the pathogen recognition pathway, is associated with lower lung function. *TLR5* is a cell surface marker which recognizes flagellin and activates proinflammatory markers. *TLR5* mediated signaling in the airway structural cells, following exposure to flagellin, triggers compartmentalized mucosal innate immunity and results in improved T-cell-mediated immunity and antibody responses^35,36^. A previous study identified flagellin as a microbial product that stimulated strong allergic responses and promoted allergic sensitization to indoor allergens, by stimulating secretion of pro-inflammatory cytokines by lung epithelial cells^23^. Another study has shown that *TLR5* signaling in the airway epithelium is important for induction of proinflammatory responses such as chemokine production in neutrophils and macrophages^37^. Consistent with these studies, the present study showed that *TLR5* gene expression levels were associated with year 30 predicted FEV_1_ and FVC values suggesting that pathogen recognition maybe an important pathway associated with lung function. We hypothesize that *TLR5* overexpression may lead to excessive proinflammatory responses in the lung that ultimately reduce lung function. Since *TLR5* was associated with lower lung function but not lung function decline over 10 years, our results suggest that *TLR5* may be important in determining early life lung function and influencing the peak lung function attained rather than impact decline in lung function among adults. This study also showed that higher levels of *CCR1*, a downstream marker in the *TLR2/TLR1* pathogen recognition pathway, was associated with faster decline of FEV_1_. Asthmatics have higher *CCR1* expression in airway smooth muscle cells as compared to healthy controls^38^ and another study found increased expression of *CCR1* positive mast cells in bronchial biopsies in asthmatic patients compared to healthy controls^39^. These studies indicate that overexpression of *CCR1* may play an important role in asthma and reduced lung function. Since *CCR1* was associated with lung function decline in our study, we hypothesize that *CCR1* may have a stronger influence on decline of lung function in adults. Thus, *TLR5* and *CCR1* may influence lung function during different timepoints and overexpression of both genes may have a synergistic effect on reducing lung function. Contrary to our hypothesis, we found that 10-year decline of FEV_1_ and FVC was slower in higher levels of *MAPK14*. This needs to be investigated further as the reasons for this finding remains unclear.

The results observed in our study suggest that only extremely high levels of the markers in the TLR pathway reduce lung function suggesting biological resilience to small changes of gene expression levels in individual biological pathways. For example, the difference in lung function between the fourth and the fifth levels of FEV_1_ and FVC at year 30 for *TLR5* was higher than the difference between the other levels. The lower values of FEV_1_ and FVC at the highest 5% levels of the markers suggests that there may be a threshold value of gene expression levels of these markers influencing the decrease in lung function. However, the higher difference between fourth and fifth levels is not observed in the analysis between decline in lung function and levels of inflammatory markers. Lower FEV_1_ and FVC, within the normal range, have been shown to be strong predictors of overall mortality and other health outcomes such as cardiovascular diseases^40–43^. Previous studies^44,45^, have also shown that decline in FEV_1_ or FVC with preserved ratio is predictive of cardiovascular disease, and that this “restrictive” condition is associated with a “Hypertrophic, high output cardiac phenotype”, while obstructive disease with declining FEV_1_/FVC ratio is associated with a “Small heart, low output phenotype”^40^. While the association between decline in FEV_1_ or FVC and chronic respiratory disease has not been evaluated, we hypothesize that lower FEV_1_ and FVC and/or faster declines in FEV_1_ or FVC, within the normal range, may represent an intermediate phase between ideal lung health and chronic respiratory disease^46^. This and other studies conducted in the CARDIA cohort, provide a foundation to develop a model that informs us of transition from normal lung health to chronic respiratory disease, and study early changes in lung function as a risk factor for pulmonary and cardiovascular health outcomes.

The strengths of the study include the long-term follow-up of the participants and the representative sample with inclusion of blacks and whites, and men and women. Assessing the markers using gene expression analysis is advantageous in cases where measurement of protein level is not possible. The limitations of the study include the timing of the measurements of lung function and the gene expression markers at different years thereby limiting our understanding of the temporal relationship between gene expression levels and lung function. Measurement of gene expression levels at more than one interval will help understanding the longitudinal relationship further. However, gene expression levels measured at year 25 were analyzed with FEV_1_ and FVC measured at year 30, aligning the temporality with our hypothesis about the effect of gene expression on lung function measures. Measurement of FEV_1_ and FVC using different methods at year 20 and year 30 (a dry rolling-seal OMI spirometer at year 20 and a portable spirometer at year 30) could have impacted the measurements. However, we have followed ATS guidelines for measurement of lung function at both times thereby minimizing the variation in lung function measurements across both visits. Gene expression levels of these inflammatory markers could be correlated with differences in cell composition such as the proportion of monocytes, T-lymphocytes etc. Since complete blood counts are not available in CARDIA at year 25, differences in cell composition may be a potential confounder in the observed association. A study evaluating the expression of Toll like receptors on peripheral blood mononuclear cells (PBMCs) showed that *TLR5* was expressed in T cells, NK cells and monocytes but was not expressed in B cells or plasmacytoid dendritic cells.^47^. In addition, *TLR5* is also expressed in airway neutrophils^48^. Thus, *TLR5* appears to be expressed in all major cell subsets in peripheral blood apart from the B cells. Thus, changes in B cell distribution may impact *TLR5* expression in peripheral blood. Lastly, we did not adjust for multiple comparisons in these analyses as these genes were selected based on a *a priori* hypothesis regarding the role of inflammatory markers in lung function. Using Bonferroni correction and a p-value of 0.003 (6 biomarkers with three outcomes = 0.05/18 = 0.0003) to determine statistical significance would result in none of the observed associations being statistically significant. Hence these results, though suggestive of an association between gene expression levels in *TLR5*, *MAPK14* and *CCR1* and lung function, need to be confirmed in independent studies.

In conclusion, the results suggest that high levels of gene expression of *TLR5 and CCR1* are associated with lower lung function and these results are independent of smoking and BMI. These results suggest that pathogen recognition pathway may be important in influencing lung health. Future studies that include measurement of gene expression levels at multiple timepoints in independent datasets need to be conducted to determine the specific genes that may be important in the longitudinal decline in lung function in healthy adults and whether modulation of the pathogen recognition pathways may be helpful in improving lung health among young adults.

## Supplementary information

Supplementary file.
