# Supplementary material for: Lung Function and Gene Expression of Pathogen Recognition Pathway Receptors: the Cardia Lung Study
Source: Sci Rep. 2020 Jun 9;10:9360. doi: 10.1038/s41598-020-65923-z (PMC7283270; doi:10.1038/s41598-020-65923-z)
Supplement: Supplementary file 1 — Supplementary file. [file 41598_2020_65923_MOESM1_ESM.docx]

**LUNG FUNCTION AND GENE EXPRESSION OF PATHOGEN RECOGNITION PATHWAY RECEPTORS: THE CARDIA LUNG STUDY**

Ramya Ramasubramanian^1^, Ravi Kalhan^2,3^, David R. Jacobs Jr.^1^, George R. Washko^4,5^, Lifang Hou^2^, Myron D. Gross^6^, Weihua Guan^7^, Bharat Thyagarajan^6,8^.

1. Division of Epidemiology and Community Health, University of Minnesota School of Public Health, Minneapolis, MN, USA
2. Department of Preventive Medicine, Northwestern University Feinberg School of Medicine, Chicago, IL, USA
3. Division of Pulmonary and Critical Care Medicine, Northwestern University Feinberg School of Medicine, Chicago, IL, USA
4. Division of Pulmonary and Critical Care Medicine, Brigham and Women’s Hospital, Boston, MA, USA
5. Applied Chest Imaging Laboratory, Brigham and Women’s Hospital, Boston, MA, USA
6. Department of Pathology and Laboratory Medicine, University of Minnesota School of Medicine, Minneapolis, MN, USA
7. Department of Biostatistics, University of Minnesota School of Public Health, Minneapolis, MN, USA

**Supplementary table 1a: Participant characteristics at year 25 with respect to *MAPK14* gene expression levels**

| Characteristics | *MAPK14* gene expression levels | | | | | p-value |
| --- | --- | --- | --- | --- | --- | --- |
|  | 0-25 percentile  (n=568) | >25-50 percentile  (n=568) | >50-75 percentile  (n=568) | >75-95 percentile  (n=454) | >95-100 percentile  (n=113) |  |
| Age (years) | 50.09  (3.68) | 49.95  (3.64) | 50.27  (3.59) | 50.26  (3.35) | 50.39  (3.43) | 0.47 |
| Race | | | | | | |
| %Blacks | 60.92 | 44.01 | 35.92 | 29.30 | 36.28 | <0.0001 |
| Sex | | | | | | |
| % Female | 57.57 | 56.87 | 56.69 | 62.11 | 64.60 | 0.22 |
| Smoking | | | | | | |
| Never | 67.96 | 66.37 | 64.08 | 62.33 | 54.87 | 0.13 |
| Former | 20.42 | 22.01 | 23.77 | 22.03 | 27.43 |  |
| Current | 11.62 | 11.62 | 12.15 | 15.64 | 17.70 |  |
| BMI | 29.85 (6.53) | 29.56  (6.88) | 29.67  (6.79) | 30.19  (7.49) | 32.22  (9.08) | 0.005 |
| Alcohol consumption (mL/day) | 10.29  (22.83) | 11.41  (17.99) | 11.39  (19.40) | 10.76  (19.54) | 9.65  (19.94) | 0.79 |
| C-reactive protein (uG/ML) | 2.45  (3.70) | 2.48  (4.41) | 2.77  (4.16) | 3.58  (5.43) | 6.42  (8.07) | <0.0001 |

**Supplementary table 1b: Participant characteristics at year 25 with respect to *CCR1* gene expression levels**

| Characteristics | *CCR1* gene expression levels | | | | | p-value |
| --- | --- | --- | --- | --- | --- | --- |
|  | 0-25 percentile  (n=568) | >25-50 percentile  (n=568) | >50-75 percentile  (n=568) | >75-95 percentile  (n=454) | >95-100 percentile  (n=113) |  |
| Age (years) | 50.31  (3.47) | 50.29  (3.60) | 50.07  (3.58) | 49.97  (3.59) | 49.67  (3.83) | 0.24 |
| Race | | | | | | |
| %Blacks | 27.64 | 38.20 | 43.84 | 57.93 | 77.88 | <0.0001 |
| Sex | | | | | | |
| % Female | 46.65 | 54.40 | 62.15 | 70.26 | 71.68 | <0.0001 |
| Smoking | | | | | | |
| Never | 64.44 | 67.08 | 67.61 | 60.35 | 59.29 | 0.005 |
| Former | 25.70 | 21.48 | 18.84 | 23.79 | 21.24 |  |
| Current | 9.86 | 11.44 | 13.56 | 15.86 | 19.47 |  |
| BMI | 28.93  (6.21) | 29.41  (6.51) | 30.22  (7.54) | 31.19  (7.76) | 30.74  (7.13) | <0.0001 |
| Alcohol consumption (mL/day) | 11.65  (16.34) | 12.15  (26.12) | 10.32  (18.23) | 9.46  (18.88) | 9.63  (13.39) | 0.18 |
| C-reactive protein (uG/ML) | 2.07  (3.35) | 2.42  (3.56) | 3.14  (5.00) | 4.03  (6.18) | 4.99  (6.36) | <0.0001 |

**Supplementary table 1c: Participant characteristics at year 25 with respect to *TLR1* gene expression levels**

| Characteristics | *TLR1* gene expression levels | | | | | p-value |
| --- | --- | --- | --- | --- | --- | --- |
|  | 0-25 percentile  (n=568) | >25-50 percentile  (n=568) | >50-75 percentile  (n=568) | >75-95 percentile  (n=454) | >95-100 percentile  (n=113) |  |
| Age (years) | 50.51  (3.56) | 50.49  (3.57) | 49.93  (3.48) | 49.75  (3.67) | 49.23  (3.36) | <0.0001 |
| Race | | | | | | |
| %Blacks | 41.37 | 38.73 | 43.84 | 45.37 | 56.64 | 0.006 |
| Sex | | | | | | |
| % Female | 51.23 | 57.57 | 59.86 | 62.78 | 74.34 | <0.0001 |
| Smoking | | | | | | |
| Never | 66.73 | 65.67 | 64.79 | 63.22 | 57.52 | 0.11 |
| Former | 22.71 | 23.06 | 22.36 | 20.26 | 24.78 |  |
| Current | 10.56 | 11.27 | 12.85 | 16.52 | 17.70 |  |
| BMI | 28.58  (6.00) | 29.12  (6.42) | 30.58  (7.39) | 31.17  (7.59) | 32.26  (8.91) | <0.0001 |
| Alcohol consumption (mL/day) | 12.11  (23.76) | 11.25  (19.37) | 10.01  (16.19) | 10.01  (20.09) | 11.21  (19.91) | 0.37 |
| C-reactive protein (uG/ML) | 1.91  (3.93) | 2.53  (3.95) | 3.25  (4.81) | 3.87  (5.49) | 5.36  (6.70) | <0.0001 |

**Supplementary table 1d: Participant characteristics at year 25 with respect to *TLR6* gene expression levels**

| Characteristics | *TLR6* gene expression levels | | | | | p-value |
| --- | --- | --- | --- | --- | --- | --- |
|  | 0-25 percentile  (n=570) | >25-50 percentile  (n=566) | >50-75 percentile  (n=568) | >75-95 percentile  (n=454) | >95-100 percentile  (n=113) |  |
| Age (years) | 50.17  (3.59) | 50.41  (3.58) | 50.13  (3.56) | 49.92  (3.55) | 49.68  (3.48) | 0.15 |
| Race | | | | | | |
| %Blacks | 46.67 | 34.45 | 40.85 | 44.93 | 68.14 | <0.0001 |
| Sex | | | | | | |
| % Female | 54.04 | 56.54 | 56.69 | 64.10 | 76.11 | <0.0001 |
| Smoking | | | | | | |
| Never | 66.32 | 69.08 | 63.03 | 60.35 | 62.83 | 0.01 |
| Former | 23.16 | 20.85 | 23.24 | 21.81 | 23.01 |  |
| Current | 10.53 | 10.07 | 13.73 | 17.84 | 14.16 |  |
| BMI | 29.51  (6.60) | 28.69  (5.95) | 29.87  (6.89) | 31.12  (7.77) | 33.45  (9.67) | <0.0001 |
| Alcohol consumption (mL/day) | 10.30  (23.43) | 11.87  (17.47) | 11.09  (19.33) | 10.52  (19.12) | 9.76  (20.57) | 0.66 |
| C-reactive protein (uG/ML) | 2.11  (3.23) | 2.38  (3.99) | 3.05  (4.66) | 4.05  (6.19) | 5.36  (6.49) | <0.0001 |

**Supplementary table 1e: Participant characteristics at year 25 with respect to *ICAM1* gene expression levels**

| Characteristics | *ICAM1* gene expression levels | | | | | p-value |
| --- | --- | --- | --- | --- | --- | --- |
|  | 0-25 percentile  (n=569) | >25-50 percentile  (n=567) | >50-75 percentile  (n=568) | >75-95 percentile  (n=454) | >95-100 percentile  (n=113) |  |
| Age (years) | 50.29  (3.54) | 50.21  (3.44) | 49.98  (3.65) | 50.09  (3.69) | 50.17  (3.55) | 0.64 |
| Race | | | | | | |
| %Blacks | 43.06 | 42.50 | 43.49 | 43.83 | 37.17 | 0.77 |
| Sex | | | | | | |
| % Female | 54.83 | 55.20 | 61.09 | 63.22 | 60.18 | 0.02 |
| Smoking | | | | | | |
| Never | 65.38 | 64.20 | 65.32 | 64.54 | 63.72 | 0.99 |
| Former | 21.97 | 22.22 | 22.36 | 22.47 | 23.89 |  |
| Current | 12.65 | 13.58 | 12.32 | 13.00 | 12.39 |  |
| BMI | 29.94  (6.84) | 30.32  (7.38) | 29.52  (6.68) | 29.92  (7.19) | 29.64  (7.48) | 0.43 |
| Alcohol consumption (mL/day) | 12.48  (26.25) | 10.52  (17.54) | 10.82  (19.12) | 9.63  (15.24) | 10.45  (16.35) | 0.23 |
| C-reactive protein (uG/ML) | 2.82  (5.24) | 2.89  (4.10) | 2.59  (4.21) | 3.49  (5.19) | 3.75  (5.60) | 0.01 |

**Supplementary table 2: Association between year 30 lung function and year 25 gene**

**expression levels**

| Year 30 % predicted FEV1 | | | | | | | | |
| --- | --- | --- | --- | --- | --- | --- | --- | --- |
| Markers | 0 to 25 percentile | 25 to 50 percentile | 50 to 75 percentile | 75 to 95 percentile | 95 to 100 percentile | Difference between lowest quartile and highest levels | p-value for trend |  |
| *TLR1* | 92.82 ± 0.63 | 93.10 ± 0.63 | 91.50 ± 0.62 | 92.53 ± 0.70 | 91.54 ± 1.40 | 1.28 (-1.75, 4.30) | 0.47 |  |
| *TLR6* | 92.16 ± 0.63 | 92.66 ± 0.63 | 92.69 ± 0.62 | 92.24 ± 0.70 | 92.18 ± 1.41 | -0.02 (-3.06, 3.01) | 0.97 |  |
| *ICAM1* | 91.88 ± 0.62 | 91.95 ± 0.62 | 92.19 ± 0.62 | 93.98 ± 0.70 | 92.69 ± 1.40 | -0.81 (-3.82, 2.20) | 0.14 |  |
| Year 30 % predicted FVC | | | | | | | | |
| *TLR1* | 94.62 ± 0.57 | 94.39 ± 0.57 | 93.69 ± 0.57 | 94.07 ± 0.63 | 94.00 ± 1.27 | 0.61 (-2.12, 3.35) | 0.53 |  |
| *TLR6* | 94.02 ± 0.57 | 94.25 ± 0.57 | 94.57 ± 0.56 | 93.75 ± 0.63 | 94.56 ± 1.27 | -0.54 (-3.28, 2.20) | 0.93 |  |
| *ICAM1* | 93.93 ± 0.56 | 93.87 ± 0.56 | 93.87 ± 0.56 | 95.55 ± 0.63 | 93.24 ± 1.27 | 0.69 (-2.03,3.41) | 0.42 |  |
| Year 30 % predicted FEV1/ % predicted FVC | | | | | | | | |
| *TLR1* | 98.13 ± 0.34 | 98.69 ± 0.34 | 97.69 ± 0.34 | 98.44 ± 0.38 | 97.32 ± 0.75 | 0.82 (0.81, 2.44) | 0.83 |  |
| *TLR6* | 98.04 ± 0.34 | 98.33 ± 0.34 | 98.06 ± 0.34 | 98.49 ± 0.38 | 97.51 ± 0.76 | 0.53 (-1.10, 2.16) | 0.96 |  |
| *ICAM1* | 97.88 ± 0.34 | 97.91 ± 0.34 | 98.24 ± 0.34 | 98.49 ± 0.38 | 99.51 ± 0.75 | -1.63 (-3.25, -0.02) | 0.14 |  |

Note: All percentages are represented as percentage ± SD. The differences are represented with the 95% CI.

**Supplementary table 3: Association between 10-year change in lung function from year 20 to year 30 and year 25 gene expression profiles**

| % predicted FEV1 – 10-year decline | | | | | | | |
| --- | --- | --- | --- | --- | --- | --- | --- |
| Markers | 0 to 25 percentile | 25 to 50 percentile | 50 to 75 percentile | 75 to 95 percentile | 95 to 100 percentile | Difference between first and final levels | p-value for trend |
| *TLR1* | 2.51±  0.39 | 2.50±  0.39 | 2.48±  0.39 | 2.69±  0.44 | 2.70±  0.88 | -0.19  (-2.09,  1.69) | 0.72 |
| *TLR6* | 3.27±  0.39 | 2.16±  0.39 | 2.43±  0.39 | 2.40±  0.44 | 1.94±  0.88 | 1.33  (-0.556,  3.22) | 0.15 |
| *ICAM1* | 2.72±  0.39 | 2.52±  0.39 | 2.76±  0.39 | 2.25±  0.44 | 1.89±  0.89 | 0.83  (-1.07,  2.74) | 0.59 |
| % predicted FVC – 10-year decline | | | | | | | |
| *TLR1* | 3.14±  0.39 | 2.88±  0.39 | 2.61±  0.39 | 2.99±  0.44 | 3.08±  0.87 | 0.07  (-1.81,  1.94) | 0.45 |
| *TLR6* | 3.88±  0.39 | 2.60±  0.39 | 2.69±  0.39 | 2.53±  0.43 | 2.17±  0.87 | 1.71  (-0.16,  3.58) | 0.06 |
| *ICAM1* | 3.06±  0.39 | 2.86±  0.39 | 3.17±  0.39 | 2.78±  0.43 | 1.57±  0.88 | 1.49  (-0.39,  3.38) | 0.49 |
| % predicted FEV1/ % predicted FVC – 10-year decline | | | | | | | |
| *TLR1* | -1.03±  0.23 | -1.04±  0.22 | -0.72±  0.22 | -0.96±  0.25 | -1.23±  0.50 | 0.19  (-0.89,  1.29) | 0.65 |
| *TLR6* | -1.03±  0.22 | -1.06±  0.23 | -0.77±  0.22 | -0.91±  0.25 | -1.03±  0.51 | 0.001  (-1.09,  1.08) | 0.74 |
| *ICAM1* | -0.79±  0.22 | -0.86±  0.22 | -1.08±  0.22 | -1.23±  0.25 | -0.35±  0.50 | -0.45  (-1.53,  0.63) | 0.99 |

Note: All percentages are represented as percentage ± SD. The differences are represented with the 95% CI.

**Figure 1: Correlation between gene expression profiles of six genes in the Toll Like Receptor pathway.**


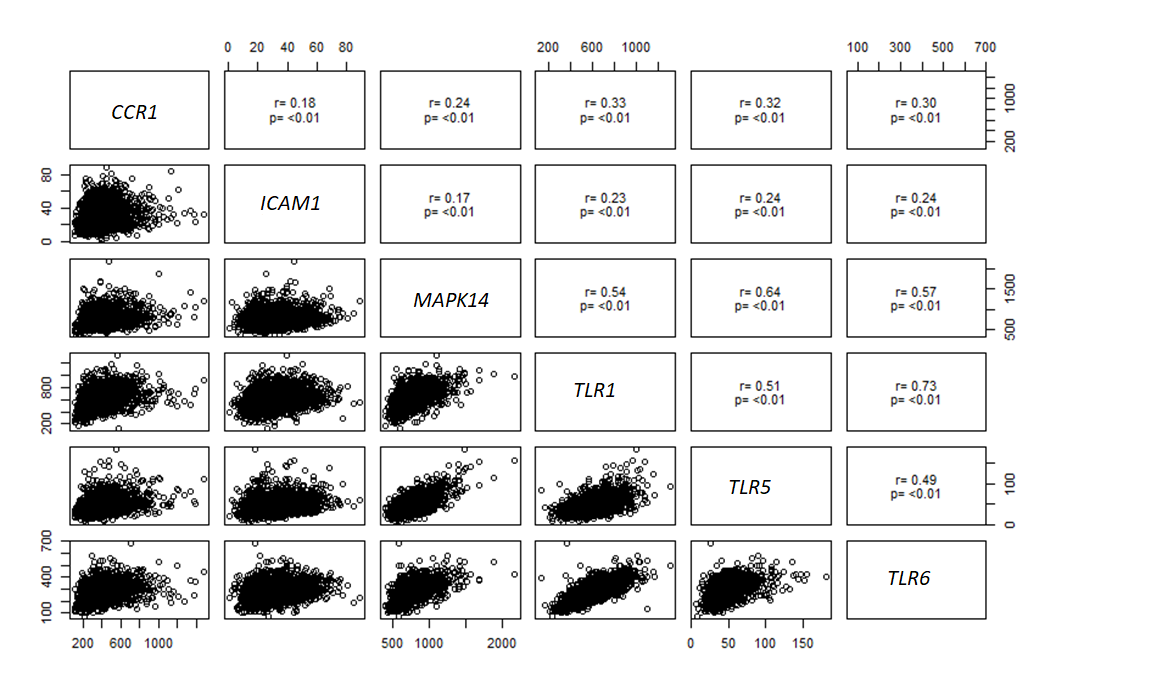


Note: r is the pearson correlation coefficient between the genes.
